# Association of polymorphisms in the FOXO1 and UCP3 genes with nonalcoholic fatty liver disease in Chinese children

**DOI:** 10.1186/1687-9856-2013-S1-O37

**Published:** 2013-10-03

**Authors:** Yan-ping Xu, Li Liang, Chun-lin Wang

**Affiliations:** 1 Department of Endocrinology, The Children's Hospital of Zhejiang University School of Medicine 310003, Zhugan Xiang, Hangzhou, Zhejiang province, China

## Aim

The human protein encoded by the FOXO1 gene functions as a transcription factor of insulin signaling key genes. Human uncoupling proteins 3 (UCP3) are mitochondrial proteins that are involved in the control of energy metabolism and the pathophysiology of obesity. In this study we investigated the role of genetic variation in the FOXO1 and UCP3 gene in susceptibility to non-alcoholic fatty liver disease (NAFLD) and relevant metabolic traits.

## Methods

We genotyped nine single nucleotide polymorphisms (SNPs) for association analyses in children (250 patients with NAFLD, 111 patients with metabolic sydrome, 146 with obese and 200 controls). Body mass index (BMI), waist and hip circumference, blood pressure, fasting blood glucose (FBG), insulin (FIN), lipid profiles were measured and performed B-ultrasound examination in all the subjects.

## Results

In the NAFLD group, FOXO1A and UCP3 allele were significantly more frequent in both association studies. There was a significant difference in the overall distribution of the genotype frequencies (UCP3 rs11235972, rs6195, rs1800849), (FOXO1 rs2721068), and there was a significant difference (P = 0.0298, 0.0191) in the distribution of the haplotype (UCP3 rs11235972, UCP3 rs1800849), might be good NAFLD markers.

## Conclusion

In conclusion, our study suggests a effect of UCP3 haplotype on NAFLD development and relevant intermediate phenotypes which predispose for NAFLD.

